# "Working Men" and Hospital Committees

**Published:** 1890-03-15

**Authors:** 


### Passing Topics.

"WORKING MEN" AND HOSPITAL COMMITTEES.

We understand that an organized effort is to be made at the next meeting of the subscribers to the Royal Hospital for Diseases of the Chest to obtain seats at the board of that institution for working men members. It will be remembered in connection with the recent dispute at this hospital that the working classes claimed a double share of interest in its affairs, first as patients and then as supporters. An examination of its accounts and its work revealed, however, that while an overwhelming proportion of the patients came from the neighbourhood of the hospital, nearly the whole of the income was derived from the City and the West End; the sum contributed by the working classes being in this case, as in most others, little more than infinitesimal.

We believe the Hospital Saturday Fund Council are responsible for the first suggestion that the working classes may rightfully claim seats at Hospital Boards in return for their contributions, and we are disposed to think that few more unfortunate propositions have been made. The objection is not to a working-man member because of his class. On the contrary, if he were elected in the constitutional manner, however unnecessary the innovation might appear, no one would have the right to object. But there is all the difference in the world between allowing this, and granting that working men, because they contribute an unappreciable something towards the total of a fund which is expended almost wholly upon their class, are therefore entitled to "representation." When certain proposals were put forward some time ago with a view to organize the help of the working classes in mitigation of the chronic deficit of the hospitals, and by means of a weekly collection of a penny apiece to infuse something of warmth and vitality into the anæmic organization which represents the concern and sympathy of those classes for the institutions which benefit them, the utmost dream of innocent enthusiasm was to raise one hundred thousand pounds. It was excusable—indeed, it was creditable—to the promoters of that scheme, including no less astute a personage than a Lord Mayor, that coming fresh and hopeful to the question, they should think the proposal practicable. Upon paper, nothing can look easier. But the result has only demonstrated what everyone who had studied the question knew before, that the means has not yet been discovered, and is apparently undiscoverable, of arousing any class of the community to a full sense of its obligation to the hospitals.

Supposing, however, the £100,000 had been forthcoming, the working men would have provided just one shilling out of every seven shillings and sixpence actually disbursed by the voluntary hospitals in their relief. But this is by no means all. In the seven shillings and sixpence nothing is reckoned for the capital sunk in hospital land and buildings, and in equipment, or for the value of unremunerated services —pre-eminently those of the physicians and surgeons. It is not straining the argument to say that though twice £100,000 were poured into the treasuries by the patients, the hospitals would still remain charitable institutions.

Of course the position thus nakedly pourtrayed will offend some susceptibilities. The propriety of allowing the poorer classes to depend upon charity to supply a necessary of life, even in stress of illness, is doubted by those whose minds are schooled to certain austere methods; while, on the other hand, some people appear to believe that a minute contribution in aid renders the recipient independent and entitled to a voice in the management. But if the working men are to justify this claim they must bestir themselves to good purpose and with a will of which they have, as yet, given no evidence. To eliminate charity from hospital work the patient class must find not one hundred thousand pounds annually, but a million at least. When they do this they may without scruple urge claims which at present seem inadmissible.

Agricola.

